# The relationship between traumatic birth perception and mother-infant bonding of women who had emergency and planned caesarean sections

**DOI:** 10.1186/s12884-026-09958-6

**Published:** 2026-09-15

**Authors:** Ayşe Çataloluk, Rumeysa Şener, Hümeyra Sağlam

**Affiliations:** Faculty of Health Sciences, Department of Midwifery, Tokat Gaziosmanpaşa University, Tokat, Türkiye

**Keywords:** Caesarean section, Childbirth, Emergencies, Postpartum period

## Abstract

This study aimed to determine the relationship between traumatic birth perception and mother-infant bonding in women who had emergency and planned caesarean. This was a descriptive, single-centre cross-sectional study conducted between January 20 and May 15, 2024, among puerperal women within the first month after birth at a university hospital. The study was completed with 182 women, 86 in the emergency caesarean group and 96 in the planned caesarean group. Approval from the ethics committee and the relevant institution was obtained before starting the study. Data were collected using a personal information form to determine the socio-demographic and descriptive characteristics of women, the “Traumatic Childbirth Perception Scale (TCPS)” and the “Mother to Infant Bonding Scale (MIBS)”. Of the women who had emergency caesarean section, 37.2% had a university degree or higher, 74.4% were unemployed, 51.2% had an unplanned pregnancy and 73.3% perceived their last birth experience as negative. Of those who had planned caesarean, 32.3% had primary school degrees, 67.7% were unemployed, 60.4% had an unplanned pregnancy and 70.8% evaluated their last birth experience as positive. A statistically significant difference was found between the mean TCPS scores of women who had emergency (X̄ = 71.74 ± 23.70) and planned (X̄ = 64.34 ± 18.63) caesarean sections (mean difference = 7.31, 95% CI: 1.10–13.51; *p* = 0.021). No statistically significant difference was found between the emergency and planned caesarean status of women and the median MIBS scores (*p* = 0.204). A negative moderately significant correlation was found between the total TCPS score and the total MIBS score of women who had planned caesarean (rₛ = − 0.417, 95% CI: − 0.585 to − 0.221; *p* < 0.001). The perception of traumatic birth in women who had emergency caesarean was higher than that of women who had planned caesarean. Among women who underwent planned caesarean sections, higher traumatic childbirth perception was associated with lower MIBS scores, corresponding to fewer reported mother–infant bonding difficulties.

**Supplementary Information:**

The online version contains supplementary material available at https://doi.org/10.1186/s12884-026-09958-6.

## Introduction

Caesarean section is among the most frequently performed surgical procedures, and many parts of the world have seen a sharp increase in caesarean section rates in recent years [6, 43]. There is a widespread belief that operative delivery is less painful and safer. This has led to an increase in the number of caesarean sections performed without medical necessity [34]. The rise in caesarean section rates leads to an increase in the number of women with a previous caesarean section. In this situation, the most appropriate birthing approach is whether to have another caesarean section, known as an elective repeat caesarean section, or to plan a vaginal birth after a previous caesarean section, known as a planned vaginal birth [32]. Caesarean section was initially a surgical solution to address the problems associated with difficult birth. However, increasing popularity has now led to a rapid growth in the number of caesarean operations around the world [12].

Emergency caesarean sections are often associated with high rates of maternal and neonatal morbidity and mortality. In such births, the time between the decision to perform a caesarean section and the actual delivery is considered an important and modifiable factor that can influence perinatal outcomes [21, 26]. Because emergency caesarean sections are performed in situations where the life of the mother or fetus is seriously threatened during labor, the decision regarding the timing of delivery should be made as soon as possible [29]. Although a significant increase in emergency and planned caesarean sections has been observed in recent years, the World Health Organization (WHO) recommends maintaining caesarean section rates between 10 and 15% [41].

Traumatic birth occurs when a woman perceives the birth process as a threat of injury or death for herself and her baby [1, 28]. Factors such as loss of control, severe pain, fetal distress, rapid labor, and postpartum hemorrhage may contribute to this perception. Furthermore, fear of childbirth, an intervention-based birth experience, inadequate pain management, concerns about epidural anesthesia, and long, difficult labor processes also contribute to negative birth experiences [2, 37].

In its 2018 recommendations, the WHO emphasized that a positive birth experience should encompass not only obstetric outcomes but also the woman's personal experience of birth. In this context, a positive birth experience is defined as a safe and supportive birth process in which the woman's beliefs, values, and expectations are taken into account [42]. Furthermore, systematic reviews reveal that perinatal outcomes depend not only on medical practices but also on holistic, high-quality care that prioritizes the woman's needs [5, 9, 42]. Practices such as skin-to-skin contact between the mother and her baby after birth and early breastfeeding have been reported to have significant effects on a positive birth experience and strengthen the mother-infant bonding [7, 15]. Mother-infant bonding is considered one of the fundamental relationships that begins to develop from the embryonic period and affects the social, emotional, and cognitive development of the individual [13, 14].

Previous studies have shown that women who undergo emergency caesarean sections report higher levels of traumatic childbirth perception than those who undergo planned caesarean sections. In addition, the mode of birth has been associated with mother–infant bonding, although this relationship appears to be influenced by individual psychosocial and obstetric factors [15, 22]. However, limited evidence is available regarding the relationship between traumatic childbirth perception and mother–infant bonding specifically among women undergoing emergency and planned caesarean sections. Furthermore, few studies have directly compared these two groups while simultaneously examining the factors associated with traumatic childbirth perception and mother–infant bonding during the early postpartum period. Addressing this gap may contribute to a better understanding of women's psychological experiences following caesarean birth and support the development of targeted postpartum care. Therefore, this study aimed to determine the relationship between traumatic childbirth perception and mother–infant bonding in women who underwent emergency and planned caesarean sections. To this end, the research questions are as follows:

- 1. Is there a significant difference between the levels of traumatic birth perception in women who undergo emergency caesarean sections and planned caesarean sections?
- 2. Is there a significant difference between the levels of mother-infant bonding in women who undergo emergency caesarean sections and planned caesarean sections?
- 3. Is there a significant relationship between women's perception of traumatic birth and mother-infant bonding?
- 4. What demographic and birth-related factors are associated with traumatic childbirth perception?

## Materials and methods

### Type and time of the research

The research employed a descriptive, cross-sectional design and was conducted between January 20 and May 15, 2024.

### Research population and sample

**Fig. 1:**
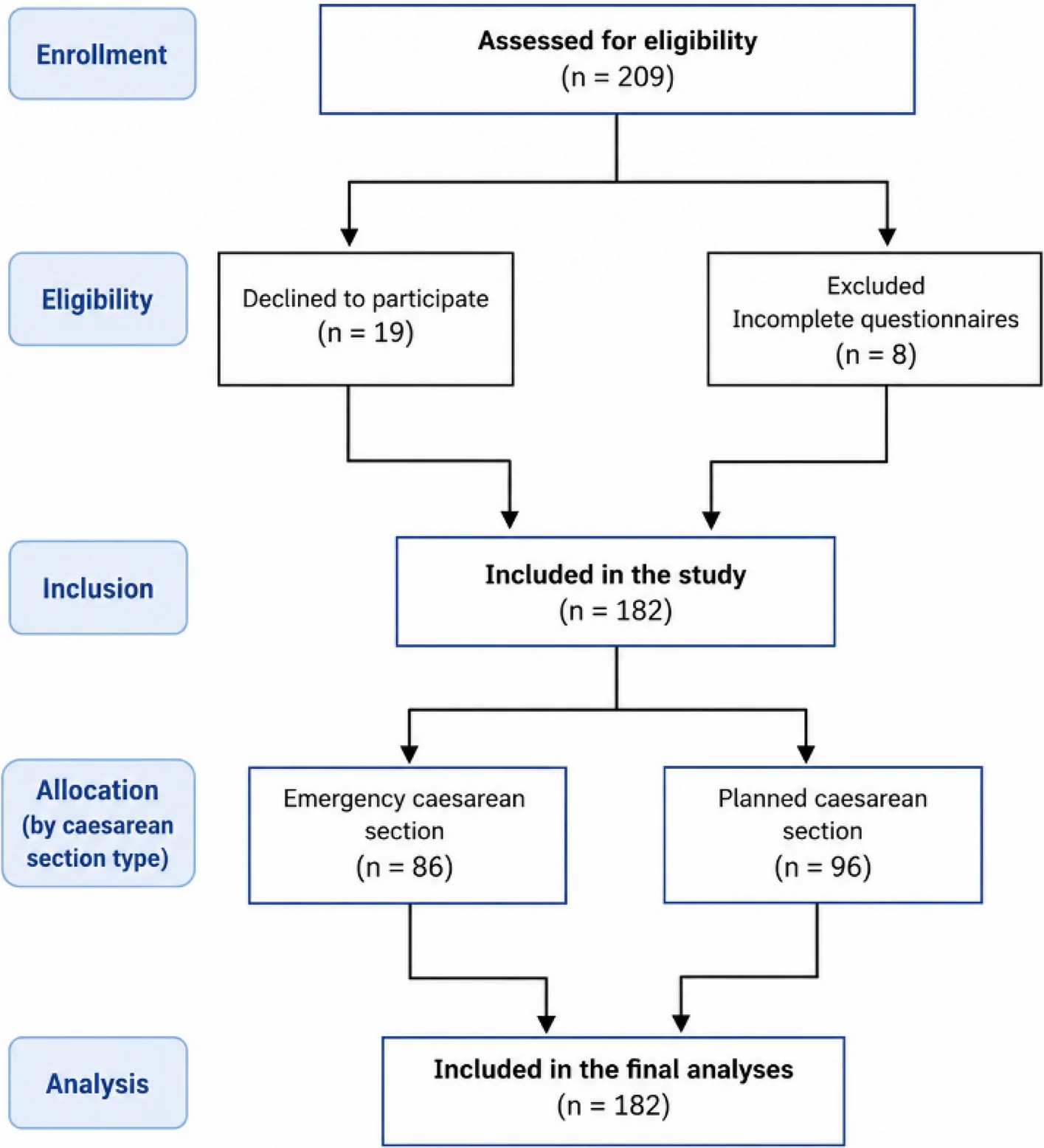
Participant flow diagram

The study population consisted of postpartum women in the obstetrics and gynecology outpatient clinics, delivery rooms, and obstetrics and gynecology wards of a university hospital in the Central Black Sea Region of Türkiye. Sample size was calculated using power analysis using G*Power 3.1.9.7 to compare the means of two independent groups to examine the difference between the perception of traumatic birth and mother-infant bonding levels of women undergoing emergency and planned caesarean sections [19]. The calculation was based on a 5% significance level (α = 0.05), 80% statistical power (1–β = 0.80), and Cohen's recommended medium effect size (d = 0.5) [10, 11]. Accordingly, a minimum of 64 participants per group (128 participants in total) was required. To account for an anticipated 10% attrition rate, the target sample size was increased to 142 participants. Women who met the eligibility criteria were invited to participate in the study on a voluntary basis during the data collection period. A total of 209 women were assessed for eligibility; 19 declined to participate, and 8 were excluded because they did not complete the study questionnaires. Consequently, the final study sample consisted of 182 women, including 86 who underwent emergency caesarean section and 96 who underwent planned caesarean section. All included participants completed the questionnaires and were included in the statistical analyses (Fig. 1).

### Inclusion and exclusion criteria

Women who voluntarily agreed to participate in the study, were at least primary school graduates, had undergone a caesarean section, were within one month postpartum, and completed the Personal Information Form, the Traumatic Childbirth Perception Scale (TCPS), and the Mother-to-Infant Bonding Scale (MIBS) were included in the study. Women younger than 18 years of age, those with multiple pregnancies, stillbirth, a diagnosed psychiatric disorder, or an inability to communicate in Turkish were excluded from the study. In addition, participants who did not complete the study questionnaires were excluded from the final analyses.

### Data collection tools

A personal information form, TCPS, and MIBS were used as data collection tools to determine the sociodemographic and descriptive characteristics of the women.

### Personal information form

The form, developed by the researchers after reviewing relevant literature [1, 17, 31, 36], included 11 questions to determine the sociodemographic and obstetric characteristics of the participants.

### TCPS

Developed by [44] to determine women's perception of traumatic birth, the scale consists of 13 items. Each item is scored from 0 to 10. The minimum score for the entire scale is 0 and the maximum is 130. Total mean scores on the scale indicate a very low level of perception of traumatic birth (0–26), low (27–52), moderate (53–78), high (79–104), and very high (105–130). The Cronbach's alpha reliability coefficient for the original scale was reported as 0.89. In this study, Cronbach's alpha was calculated as 0.92.

### MIBS

Developed by [38], it measures the continuous development of bonding between mother and baby in the first 12 weeks after birth. Thanks to this feature, the scale is suitable for both evaluating the early postpartum period and for re-examining bonding in short time intervals. The adaptation, validity, and reliability studies of the scale in Türkiye were conducted by Karakulak-Aydemir and Alparslan [3]. MIBS is a scale consisting of a total of 8 items, each scored with 4-point Likert-type response options. The answers take a value between 0 and 3, and scale scores range from 0 to 24. Higher scores indicate greater problems experienced in mother-infant bonding. Since items 1, 4, and 6 of the scale include positive emotional expressions, they are scored as 0, 1, 2, and 3; Items 2, 3, 5, 7, and 8 are negatively worded, so they are reverse-scored as 3, 2, 1, or 0. The internal consistency reliability (Cronbach's Alpha) of the scale was reported as 0.66 [3]. In this study, Cronbach's Alpha was found to be 0.72.

### Data collection

Forms were distributed to postpartum women who agreed to participate in the study, and the forms were completed by the postpartum women themselves, under the supervision of the researchers, within an average of 10–15 min. Before completing the study questionnaires, participants had not received any structured psychological support, postpartum debriefing, or other psychological intervention.

### Data analysis

Statistical analyses were conducted using the SPSS 22.0 package program. The analytical approach was selected according to the specific aims of the study. Comparative analyses were performed to determine whether traumatic childbirth perception and mother–infant bonding differed between women undergoing emergency and planned caesarean sections and according to sociodemographic and obstetric characteristics. Correlation analysis was used to examine the relationship between traumatic childbirth perception and mother–infant bonding.

Descriptive statistics were presented as number (n), percentage (%), mean ± standard deviation (SD), or median (Q1–Q3), as appropriate. The Kolmogorov–Smirnov test was used to evaluate the distribution of continuous variables. Normally distributed variables were compared using the independent-samples t test (two groups) or one-way ANOVA (three or more groups), followed by Tukey's post-hoc test when appropriate. Non-normally distributed variables were compared using the Mann–Whitney U test (two groups) or the Kruskal–Wallis test (three or more groups). When the Kruskal–Wallis test was significant, post-hoc pairwise comparisons were performed using Mann–Whitney U tests with Bonferroni correction. Spearman's rank correlation coefficient was used to examine associations between non-normally distributed variables. The 95% confidence intervals for Spearman's rank correlation coefficients were estimated using bootstrap resampling. The difference between the Spearman correlation coefficients of the emergency and planned caesarean section groups was also evaluated using bootstrap resampling, with a 95% confidence interval calculated for the difference between the coefficients. Two-way ANOVA was performed only for TCPS scores, which met the assumptions for parametric analysis, to examine the main effects of caesarean section type (emergency versus planned) and sociodemographic and obstetric characteristics, as well as their interaction. Since MIBS scores did not meet the assumption of normal distribution, bivariate group comparisons involving MIBS were conducted using non-parametric methods.

Separate multiple linear regression analyses were performed using the enter method. The regression models included age, educational level, employment status, pregnancy planning, parity, previous mode of birth, gestational age, and perception of the most recent birth experience as potential predictors. Results were evaluated at a 95% confidence interval, and *p* < 0.05 was considered statistically significant.

### Ethical aspects of the research

Ethical approval was obtained from the Ethics Committee for Social and Human Sciences Research at Tokat Gaziosmanpaşa University before commencing the research (dated December 26, 2023, numbered 01–68). Written permission was also obtained from the hospital where the research was conducted (dated January 18, 2024, numbered E-50498206–044–387825). Verbal and written consent were obtained from the participants who volunteered to participate in the study. The research was conducted in accordance with the principles of the Declaration of Helsinki.

## Results

The study found that the mean age of women undergoing emergency caesarean section was 29.03 ± 6.56 (18–44) years, 37.2% had a university degree or higher, 74.4% were unemployed, 51.2% had an unplanned pregnancy, 62.8% were multiparous, 37.2% had never given birth before, 73.3% had a gestational age of < 37 weeks, and 73.3% perceived their last birth experience as negative. It was determined that the mean age of women who had a planned caesarean section was 32.01 ± 6.28 (18–46) years, 32.3% were primary school graduates, 67.7% were unemployed, 60.4% had an unplanned pregnancy, 78.1% were multiparous, 37.5% had a previous caesarean delivery, 90.6% had a gestational age of 37 weeks or more, and 70.8% perceived their last birth experience as positive (Table 1).

**Table 1:** Comparison of Socio-Demographic and Obstetric Characteristics of Women

Characteristics		Emergency caesarean (n = 86)		Planned caesarean(n = 96)		Test value/p
		**Number (n)**	**Percentage (%)**	**Number (n)**	**Percentage (%)**	
**Age**	18–30	73	84.9	64	66.7	***χ2* = *7.139***
	31 +	13	15.1	32	33.3	***p* = *0,008***
**Mean of age X̄ ± SD (min–max)**		29.03 ± 6.56 (18–44)		32.01 ± 6.28 (18–46)		***t* = *−3.122***
						***p* = *0.002***
**Education**	Primary school	24	27.9	31	32.3	
	High school	30	34.9	38	29.6	χ2 = 1.712
	University degree or	32	37.2	27	28.1	p = 0.425
	higher					
**Employment**	Yes	22	25.6	31	32.3	χ2 = 0.990
	No	64	74.4	65	67.7	p = 0.320
**Pregnancy planning**	Yes	42	48.8	38	39.6	χ2 = 1.577
	No	44	51.2	58	60.4	p = 0.209
**Mean of pregnancies** **X̄ ± SD (min–max)**		2.27 ± 1.29 (1–6)		2.65 ± 1.32 (1–6)		t = −1.933
						p = 0.055
**Parity**	Primiparous	32	37.2	21	21.9	***χ2* = *5.168***
	Multiparous	54	62.8	75	78.1	***p* = *0.023***
**Mean of parity** **X̄ ± SD (min–max)**		1.97 ± 1.02 (1–6)		2.34 ± 1.10 (1–6)		***t* = *−2.313***
						***p* = *0.022***
**Previous birth type**	Normal	17	19.8	17	17.7	χ2 = 6.648
	Caesarean	21	24.4	36	37.5	p = 0.084
	Both normal	16	18.6	22	22.9	
	caesarean	32	37.2	21	21.9	
	Non-birth					
**Gestational age**	< 37 weeks	63	73.3	9	9.4	***χ2* = *77.421***
	37 weeks and higher	23	26.7	87	90.6	***p* < *0.001***
**Mean of gestational age X̄ ± SD (min–max)**		36.23 ± 2.17 (29–39)		38.03 ± 0.60 (36–40)		***t* = *−7.634*** ***p* < *0.001***
**Baby's birth weight(gr)**		2686.45 ± 567.07 (1335–4400)		3158.44 ± 384.29 (2440–4340)		***t* = *−6.497*** ***p* < *0.001***
**Perception of the last birth**	Positive	23	26.7	68	70.8	***χ2* = *35.271***
	Negative	63	73.3	28	29.2	***p* < *0.001***

*χ2* Chi-square test, *t* Independent Sample t test, X̄ mean value, *SD* Standard Deviation

A statistically significant difference was found between the women participating in the study's emergency and planned caesarean section status and their age, parity, gestational age, baby's birth weight, and perception of the last birth (*p* < 0.05). There was no statistically significant difference between the women's emergency and planned caesarean section status and variables such as education, employment, pregnancy planning, number of pregnancies, or previous mode of delivery (*p* > 0.05), and the groups were homogeneously distributed (Table 1).

The relationship between the TCPS total scores and the sociodemographic and obstetric characteristics of women is presented in Table 2. Accordingly, a statistically significant difference was found between the age, education, parity, previous birth type, and perception of last birth experience and the mean TCPS total score of women who had emergency caesarean sections (*p* < 0.05). A statistically significant difference was found between the age, education level, employment, pregnancy planning, number of births, previous birth type, gestational age, perception of last birth experience, and the mean TCPS total score of women who had planned caesarean sections (*p* < 0.05).

**Table 2:** Comparison of the Mean TCPS of Women Who Had Emergency and Planned Caesarean Sections According to Sociodemographic and Obstetric Characteristics

Characteristics		Emergency caesarean (*n* = 86)	Planned caesarean(*n* = 96)	Test value/p
		**X̄ ± SD**	**X̄ ± SD**	
**Age**	18–30	75.06 ± 23.56 53.07 ± 14.24	71.32 ± 18.04 50.65 ± 10.42	***15.102/* < *0.001***
	31 +			
**t/p**		***3.251/0.002***	***7.096/* < *0.001***	
**Education**	Primary school^a^	56.89 ± 21.48 89.83 ± 17.64 65.93 ± 19.59	55.09 ± 17.17 62.21 ± 17.92 78.29 ± 12.68	***16.644/* < *0.001***
	High school^b^			
	University degree or higher^c^			
**F/p** **significant difference**		***21.314/* < *0.001*** ***b-a, b-c***	***15.071/* < *0.001*** ***c-a, c-b***	
**Employment**	Yes	72.72 ± 15.08 71.40 ± 26.10	74.80 ± 16.71 59.49 ± 17.54	***5.771/* < *0.001***
	No			
**t/p**		0.288/0.774	***4.059/* < *0.001***	
**Pregnancy planning**	Yes	69.26 ± 21.61 74.11 ± 25.55	70.07 ± 17.69 60.74 ± 18.45	***3.738/0.012***
	No			
**t/p**		−0.952/0.344	***2.464/0.016***	
**Parity**	Primiparous	83.37 ± 25.12 64.85 ± 20.03	81.42 ± 17.13 59.68 ± 16.18	***15.328/* < *0.001***
	Multiparous			
**t/p**		***3.765/* < *0.001***	***5.375/* < *0.001***	
**Previous birth type**	Normal^a^	70.35 ± 20.57 69.09 ± 19.10 53.43 ± 16.80 83.37 ± 25.12	50.17 ± 9.76 67.94 ± 14.75 53.50 ± 16.02 81.42 ± 17.13	**10.613/ < 0.001**
	Caesarean^b^			
	Both normal caesarean Non-birth^d^			
**F/p** **significant difference**		***7.123/* < *0.001*** ***d-c***	***18.923/* < *0.001*** ***b-a, b-c, d-a, d-b, d-c***	
**Gestational age**	< 37 weeks	72.25 ± 26.00 70.34 ± 16.18	46.33 ± 10.89 66.31 ± 18.30	***4.397/0.005***
	37 weeks and higher			
**t/p**		0.405/0.687	***−4.839/* < *0.001***	
**Perception of the last birth**	Positive	59.56 ± 25.55 76.19 ± 21.52	68.76 ± 17.02 53.92 ± 18.48	***9.466/* < *0.001***
	Negative			
**t/p**		***−3.013/0.003***	***3.785/* < *0.001***	
**Scale**				
**TCPS** **t/p**	**X̄ ± SD (min–max)**	71.74 ± 23.70 (5–116)	64.34 ± 18.63 (31–109)	***2.323/0.021***

*F* One Way ANOVA (Tukey test in posthoc analysis), *t* Independent sample t test, *Two Way ANOVA, X̄: mean value, *SD* Standard Deviation, a, b, c, d: Further analysis results regarding the cause of the difference

The results of two-way ANOVA indicated that age, education, employment status, pregnancy planning, parity, previous birth type, gestational age, and perception of the last birth experience were significantly associated with TCPS scores (*p* < 0.05; Table 2).

Comparison of the mean TCPS scores of women who underwent emergency and planned caesarean sections is presented in Table 2. A statistically significant difference was found between the mean TCPS scores of the two groups (mean difference = 7.31, 95% CI: 1.10–13.51; *p* = 0.021). The effect size was small to moderate (Cohen's d = 0.35, 95% CI: 0.05–0.64). Both groups had mean TCPS scores corresponding to a moderate level of traumatic childbirth perception; however, women who underwent emergency caesarean section had significantly higher TCPS scores (71.74 ± 23.70) than those who underwent planned caesarean Sect. (64.34 ± 18.63).

The relationship between the median MIBS total scores and the socio-demographic and obstetric characteristics of women is presented in Table 3. Accordingly, a statistically significant difference was found between the pregnancy planning status and perception of recent birth experience of women who had emergency caesarean sections and the median MIBS total score (*p* < 0.05). A statistically significant difference was found between the age, education level, employment, pregnancy planning, parity, previous birth type, gestational age, and recent birth experience variables and the median MIBS scores of women who had planned caesarean sections (*p* < 0.05).

**Table 3:** Comparison of MIBS Median Scores of Women Who Had Emergency and Planned Caesarean Sections According to Sociodemographic and Obstetric Characteristics

Characteristics		Emergency caesarean (n = 86)	Planned caesarean(n = 96)
		**Median (Q1-Q3)**	**Median (Q1-Q3)**
**Age**	18–30 age	1.00 (0.00–1.00)	0.00 (0.00–1.00)
	31 +	3.00 (0.00–4.00)	3.00 (0.00–4.00)
**Z/p**		−1.752/0.080	***−4.038/*** ** < ** ***0.001***
**Education**	Primary school^a^	0.50 (0.00–4.00)	3.00 (0.00–4.00)
	High school^b^	1.00 (1.00–3.00)	1.00 (1.00–1.00)
	University degree or higher^c^	1.00 (0.00–2.00)	0.00 (0.00–1.00)
**KW/p** **significant difference**		1.715/0.424	***20.829/*** **<** ***0.001*** ***a-b, a-c***
**Employment**	Yes	1.00 (0.00–3.00)	0.00 (0.00–1.00)
	No	1.00 (0.00–3.00)	1.00 (0.00–4.00)
**Z/p**		−0.175/0.861	***−3.975/*** ** < ** ***0.001***
**Pregnancy planni**	Yes	0.00 (0.00–2.00)	0.00 (0.00–1.00)
	No	1.00 (1.00–3.75)	1.00 (1.00–4.00)
**Z/p**		***−3.322/*** ** <** ***0.001***	***−2.553/0.011***
**Parity**	Primiparous	1.00 (0.00–1.75)	0.00 (0.00–1.00)
	Multiparous	1.00 (0.00–3.00)	1.00 (1.00–3.00)
**Z/p**		−1.447/0.148	***−2.477/0.013***
**Previous birth type**	Normal^a^	1.00 (0.00–3.50)	3.00 (0.50–4.00)
	Caesarean^b^	2.00 (1.00–3.00)	0.00 (0.00–1.00)
	Both normal caesarean^c^	0.50 (0.00–3.00)	1.00 (0.00–4.00)
	Non-birth^d^	1.00 (0.00–1.75)	0.00 (0.00–1.00)
**KW/p** **significant difference**		3.947/0.267	***18.463/*** **<** ***0.001*** ***a-b, a-d, b-c, c-d***
**Gestational age**	< 37 weeks	1.00 (0.00–3.00)	4.00 (0.50–4.00)
	37 weeks and higher	1.00 (0.00–2.00)	1.00 (0.00–1.00)
**Z/p**		−1.240/0.215	***−2.402/0.016***
**Perception of the last birth**	Positive	0.00 (0.00–1.00)	0.50 (0.00–1.00)
	Negative	1.00 (0.00–4.00)	3.00 (0.00–4.00)
**Z/p**		***−2.309/0.021***	***−2.789/0.005***
**Scale**			
**MIBS** **Z/p**	**Median (Q1-Q3)**	1.00 (0.00–3.00)	1.00 (0.00–3.00)

Data that do not show a normal distribution are presented as medians (Q1-Q3). KW = Kruskal Wallis test; Z = Mann Whitney U test value. Pairwise comparisonswere performed using Mann–Whitney U tests with Bonferroni correction

In comparisons of MIBS scores according to sociodemographic and obstetric characteristics, significant differences were observed for age, educational level, employment status, pregnancy planning, parity, previous mode of birth, gestational age, and perception of the most recent birth experience (Table 3).

Comparison of median MIBS scores of women who underwent emergency and planned caesarean sections is presented in Table 3. No statistically significant difference was found between the women's emergency and planned caesarean section status and the median MIBS scores (*p* = 0.204) (Table 3).

A statistically significant moderate negative correlation was found between total TCPS and MIBS scores among women who underwent planned caesarean section (r_s_ = − 0.417, 95% CI: − 0.585 to − 0.221; *p* < 0.001). In contrast, the correlation was not statistically significant in the emergency caesarean section group (r_s_ = 0.133, 95% CI: − 0.110 to 0.363; *p* = 0.224) (Table 4). The between-group difference in Spearman correlation coefficients was Δrₛ = 0.549 (bootstrap 95% CI: 0.240 to 0.836). As the confidence interval did not include zero, the association between TCPS and MIBS scores differed significantly between the emergency and planned caesarean section groups.

**Table 4:** Inter-scale correlation analyses

Analysis	Caesarean section group	rₛ	95% CI	p
TCPS Total – MIBS Total	Emergency caesarean	0.133	− 0.110 to 0.363	0.224
TCPS Total – MIBS Total	Planned caesarean	− 0.417	− 0.585 to − 0.221	** < 0.001**

*rs* Spearman correlation coefficient, *CI* Confidence interval, 95% CIs for Spearman’s correlation coefficients were estimated using bootstrap resampling

To identify the predictors of traumatic childbirth perception, separate multiple linear regression analyses were performed for the emergency and planned caesarean section groups. The regression models demonstrated good overall fit for both groups (emergency caesarean: R = 0.565, R^2^ = 0.319, F = 7.507, *p* < 0.001; planned caesarean: R = 0.703, R^2^ = 0.494, F = 10.617, *p* < 0.001). The variables included in the models explained 31.9% of the variance in traumatic childbirth perception among women who underwent emergency caesarean sections and 49.4% among those who underwent planned caesarean sections. In the emergency caesarean section group, age and perception of the last birth were significant predictors of traumatic childbirth perception. In the planned caesarean section group, age, employment, parity, and perception of the last birth were significant predictors of traumatic childbirth perception (Table 5).

**Table 5:** Multiple Linear Regression Analysis of Predictors of Traumatic Childbirth Perception and Mother–Infant Bonding in the Emergency and Planned Caesarean Section Groups

Independent Variables for TCPS	Β	SE	p	95% CI		Model Fit
				**Lower**	**Upper**	
**Emergency caesarean**						
Age	−22.728	6.874	***0.001***	−36.408	−9.049	R = 0.565 R^2^ = 0.319 F = 7.507 ***p* < *0.001***
Education	−2.034	2.899	0.485	−7.803	3.734	
Parity	−17.646	9.896	0.078	−37.339	2.047	
Previous birth type	−2.177	3.916	0.580	−9.971	5.616	
Perception of the last birth	16.237	5.575	***0.005***	5.142	27.332	
**Planned caesarean**						
Age	−10.594	3.728	***0.006***	−18.003	−3.185	R = 0.703 R^2^ = 0.494 F = 10.617 ***p* < *0.001***
Education	2.843	2.425	0.244	−1.978	7.663	
Employment	−8.154	3.576	***0.025***	−15.262	−1.046	
Pregnancy planning	5.984	3.551	0.096	−1.074	13.042	
Parity	−15.357	6.644	***0.023***	−28.562	−2.152	
Previous birth type	−1.758	2.678	0.513	−7.082	3.566	
Gestational age	10.060	5.205	0.057	−0.286	20.406	
Perception of the last birth	−8.655	3.840	***0.027***	−16.287	−1.023	

**Independent Variables for MIBS**	**Β**	**SE**	**p**	**95% CI**		**Model Fit**
				**Lower**	**Upper**	
**Emergency caesarean**						
Pregnancy planning	1.030	0.338	***0.003***	0.358	1.702	R = 0.389 R^2^ = 0.152 F = 7.413 ***p* = *0.001***
Perception of the last birth	0.972	0.382	***0.013***	0.213	1.731	
**Planned caesarean**						
Age	0.959	0.329	***0.005***	0.305		R = 0.666 R^2^ = 0.443 F = 8.666 ***p* < *0.001***
Education	−0.519	0.214	***0.017***	−0.945	−0.094	
Employment	0.697	0.315	***0.030***	0.070	1.324	
Pregnancy planning	0.032	0.313	0.918	−0.0590	0.655	
Parity	−0.045	0.586	0.939	−1.209	1.120	
Previous birth type	0.116	0.236	0.624	−0.353	0.586	
Gestational age	−0.561	0.459	0.225	−1.474	0.351	
Perception of the last birth	0.662	0.339	0.054	−0.011	1.335	

Multiple linear regression analysis was performed

Separate multiple linear regression analyses were also conducted to identify the predictors of mother–infant bonding. The regression models were statistically significant for both emergency caesarean (R = 0.389, R^2^ = 0.152, F = 7.413, *p *= 0.001) and planned caesarean (R = 0.666, R^2^ = 0.443, F = 8.666, *p* < 0.001) groups. The variables included in the models explained 15.2% of the variance in mother–infant bonding among women who underwent emergency caesarean sections and 44.3% among women who underwent planned caesarean sections. In the emergency caesarean section group, pregnancy planning and perception of the last birth were significant predictors of mother–infant bonding. In the planned caesarean section group, age, education, and employment were significant predictors of mother–infant bonding (Table 5).

## Discussion

The present study compared traumatic childbirth perception and mother–infant bonding between women who underwent emergency and planned caesarean sections. Women in the emergency caesarean section group reported significantly higher traumatic childbirth perception scores than those in the planned caesarean section group, whereas no significant difference was found in mother–infant bonding scores between the two groups. Furthermore, a moderate negative correlation between traumatic childbirth perception and mother–infant bonding was identified only among women who underwent planned caesarean sections. In addition, multiple linear regression analyses demonstrated that the independent predictors of traumatic childbirth perception and mother–infant bonding differed between emergency and planned caesarean section groups.

This study examined the relationship between the perception of traumatic birth and mother-infant bonding in women who delivered by emergency and planned caesarean sections. Caesarean section rates in Türkiye are significantly higher than the global average, and an increase in emergency caesarean sections, in particular, may be associated with increased psychological stress and negative experiences experienced by women during labor [26]. The high caesarean section rate of 61.5% in our country suggests a high prevalence of caesarean deliveries, which warrants further investigation into the underlying medical and non-medical factors [30]. The findings of the study showed that women who had emergency caesarean sections had a higher perception of traumatic birth than those who had planned caesarean sections. Although the difference was statistically significant, the small-to-moderate effect size indicates that its clinical importance should be interpreted cautiously. This result is consistent with the literature because emergency caesarean section involves sudden and unexpected interventions during the birth process, which can lead to greater loss of control, anxiety, panic, stress, and a negative perception of the experience in women [4, 16, 22, 23]. On the other hand, no statistically significant difference was observed in mother–infant bonding scores between women who underwent emergency and planned caesarean sections. This finding suggests that, in the present study, caesarean section type alone was not associated with differences in mother–infant bonding. Although several sociodemographic and obstetric characteristics were found to be associated with mother–infant bonding in the regression analyses, additional psychological and social factors that were not assessed in this study may also contribute to bonding outcomes and should be explored in future research. It is also reported in the literature that mother-infant bonding is closely related to the birth experience, as well as the mother's mental health, social environment, and postpartum support systems [15, 18]. Therefore, the importance of psychosocial support services provided in the postpartum period is once again highlighted.

The findings of this study revealed significant relationships between women's perception of traumatic birth and their sociodemographic and obstetric characteristics. Variables such as age, education, parity, previous birth type, and perception of last birth experience were found to be significantly associated with TCPS scores in women undergoing emergency caesarean sections. In the planned caesarean section group, in addition to these variables, employment, whether the pregnancy was planned, and week of gestation were also predictive of the TCPS. These findings suggest that traumatic childbirth perception may be associated not only with the mode of birth but also with women's prenatal experiences, sociodemographic characteristics, and cognitive appraisals related to childbirth. Similarly, the literature reports that low education level, previous negative birth experiences, type of birth, and unplanned pregnancies negatively affect women's perceptions of the birth process, increasing their traumatic birth experiences and/or fear of childbirth [24, 25, 35, 39].

In our study, we found that the median MIBS total scores differed significantly between women undergoing emergency and planned caesarean sections based on sociodemographic and obstetric characteristics. In the emergency caesarean section group, pregnancy planning and perception of the most recent birth experience were significantly associated with MIBS scores. In the planned caesarean section group, age, education, employment, the planning of the pregnancy, parity, previous birth type, gestational age, and the perception of the last birth experience were significantly associated with mother–infant bonding. These results are consistent with previous studies showing that psychosocial and obstetric characteristics are associated with mother–infant bonding [8, 15, 34]. These findings highlight the importance of personalized psychosocial interventions to support the mother-infant relationship based on the mode of birth and pregnancy conditions.

In our study, a moderately significant negative correlation was found between the total TCPS scores and the total MIBS scores of women who underwent a planned caesarean section. In this study, a moderate negative association was found between traumatic childbirth perception and mother–infant bonding scores among women who underwent planned caesarean sections. Since higher MIBS scores indicate greater difficulties in mother–infant bonding, the observed negative correlation indicates that women with higher traumatic childbirth perception reported lower MIBS scores, corresponding to fewer reported bonding difficulties. This unexpected finding should be interpreted with caution, as it may reflect the complex nature of postpartum psychological adaptation, response patterns, or other unmeasured psychosocial factors. Given the cross-sectional design of the study, no causal interpretation can be made. Further longitudinal studies are needed to clarify the relationship between traumatic childbirth perception and mother–infant bonding. It is frequently reported in the literature that intense stress, anxiety, and fear experienced during the birth process carry over into the postpartum period and negatively affect mother-baby interaction [20, 27, 33, 40]. These findings indicate that the relationship between traumatic childbirth perception and mother–infant bonding may be more complex than expected and warrants further investigation.

## Limitations

This study has several limitations. First, the cross-sectional design does not allow for causal inferences between traumatic childbirth perception and mother-infant bonding. Second, the study was conducted in a single center with a relatively limited sample size, which may restrict the generalizability of the findings. Third, the data were collected using self-report instruments, which may be subject to recall bias and social desirability bias. Despite these limitations, the study provides valuable insights into the relationship between traumatic birth perception and mother-infant bonding among women who underwent emergency and planned caesarean sections. In addition, potentially important psychosocial factors, such as postpartum depression, anxiety, and perceived social support, were not assessed and may have influenced the observed associations.

## Conclusion

This single-center cross-sectional study suggests that the mode of caesarean delivery may be an important consideration when evaluating women's traumatic childbirth perceptions in the postpartum period. Women who underwent emergency caesarean sections reported higher traumatic childbirth perception scores compared with those who underwent planned caesarean sections, whereas caesarean section type was not associated with differences in mother–infant bonding scores in this study population. These findings highlight the importance of providing individualized postpartum care that considers women's birth experiences and psychological needs following caesarean delivery. However, because the findings are based on cross-sectional data from a single-center sample, causal relationships cannot be established. Further longitudinal studies are needed to clarify how traumatic childbirth perceptions and mother–infant bonding develop over time.

## Supplementary Information

Supplementary Material 1.

## Data Availability

The dataset is available from the corresponding author upon reasonable request. The dataset supporting the conclusions of this research can be obtained from the corresponding author upon reasonable request. 1. Access to the data is subject to the following guidelines: Interested researchers must provide a comprehensive application, clearly stating the purpose of their research, how they intend to use the data, and a declaration of confidentiality. 2. The data will be made available strictly for the intended academic use described in the application. It may not be utilized for commercial gain or for any form of unauthorized secondary analysis. 3. Proper citation of this study is required in any subsequent publications or presentations based on the shared data. Details for data access: Repository: author's archive Contact for data request: [ayse.mete@gop.edu.tr, Asst. Prof. Ayşe ÇATALOLUK].

## References

[1] Aktaş S. Multigravidas’ perceptions of traumatic childbirth: its relation to some factors, the effect of previous type of birth and experience. Med Sci. 2018;7(1):203–9. 10.5455/medscience.2017.06.8728.

[2] Andersen LB, Melvaer LB, Videbech P, Lamont RF, Joergensen JS. Risk factors for developing post‐ traumatic stress disorder following childbirth: a systematic review. Acta Obstet Gynecol Scand. 2012;91(11):1261–72. 10.1111/j.1600-0412.2012.01476.x. 22670573

[3] Aydemir Karakulak H, Alpar Ö. Adaptation of Mother to Infant Bonding Scale to The Turkish Society: Aydın Sample. J Contemp Med. 2016; 6(3): 188–199. 10.16899/ctd.45668

[4] Bay F, Sayiner FD. Perception of traumatic childbirth of women and its relationship with postpartum depression. Women Health. 2021;61(5):479–89. 10.1080/03630242.2021.1927287. 33980127

[5] Bellerose M, Rodriguez M, Vivier,PM. A systematic review of the qualitative literature on barriers to high-quality prenatal and postpartum care among low-income women. Health services research. 2022;57(4):775–85. 10.1111/1475-6773.14008. PMC9264457 35584267

[6] Betran Betran AP, Ye J, Moller A-B, Zhang J, Gulmezoglu AM, Torloni MR. The increasing trend in caesarean section rates: global, regional and national estimates: 1990-2014. PLoS ONE. 2016;11(2):e0148343. 10.1371/journal.pone.0148343. PMC4743929 26849801

[7] Brubaker LH, Paul IM, Repke JT, Kjerulff KH. Early maternal-newborn contact and positive birth experience. Birth. 2019;46(1):42–50. 10.1111/birt.12378. PMC6377287 30144141

[8] Cakir D, Alparslan Ö. The investigation of the effects of the birth type variable on the mother-infant interaction and mother's perception of her the infant. J Contemp Med. 2018; 8(2), 139–147. 10.16899/gopctd.418653

[9] Cibralic S, Pickup W, Diaz AM, Kohlhoff J, Karlov L, Stylianakis A, et al. The impact of midwifery continuity of care on maternal mental health: a narrative systematic review. Midwifery. 2023;116:103546. 10.1016/j.midw.2022.103546. 36375410

[10] Cohen J. Statistical power analysis for the behavioral sciences. 2nd ed. Lawrence Erlbaum Associates; 1988.

[11] Cohen J. A power primer. Psychol Bull. 1992;112(1):155–9. 10.1037/0033-2909.112.1.155. 19565683

[12] Curran EA, Khashan AS, Dalman C, Kenny LC, Cryan JF, Dinan TG, et al. Obstetric mode of delivery and attention-deficit/hyperactivity disorder: a sibling-matched study. Int J Epidemiol. 2016;45(2):532–42. 10.1093/ije/dyw001. 27063604

[13] Daglar G, Nur N. Level of mother-baby bonding and influencing factors during pregnancy and postpartum period. Psychiatr Danub. 2018;30(4):433–40. 10.24869/psyd.2018.433. 30439803

[14] de Waal N, Boekhorst MGBM, Nyklíček I, Pop VJM. Maternal–infant bonding and partner support during pregnancy and postpartum: associations with early child social-emotional development. Infant Behav Dev. 2023;72:101871. 10.1016/j.infbeh.2023.101871. 37544195

[15] Döblin S, Seefeld L, Weise V, Kopp M, Knappe S, Asselmann E, Martini J, Garthus-Niegel S. The impact of mode of birth on parent–infant bonding and the mediating role of birth experience: Findings from the DREAM study. BMC Pregnancy and Childbirth. 2023; 23(285). 10.1186/s12884-023-05611-8 PMC10127505 37098555

[16] Döner Şİ, Kul Uçtu A. Examining the Factors Causing the Perception of Traumatic Birth. Gumushane University Journal of Health Science. 2024; 13(1): 203–215. 10.37989/gumussagbil.1320030

[17] Elmir R, Schmied V, Wilkes L, Jackson D. Women’s perceptions and experiences of a traumatic birth: a meta-ethnography. J Adv Nurs. 2010;66(10):2142–53. 10.1111/j.1365-2648.2010.05391.x. 20636467

[18] Engin N, Ayyıldız T. The investigation of mother-baby attachment based on maternal perception and some variables. Journal of Adnan Menderes University Health Sciences Faculty. 2021; 5(3): 583–596. 10.46237/amusbfd.947004

[19] Faul F, Erdfelder E, Lang AG, Buchner A. G*Power 3: a flexible statistical power analysis program for the social, behavioral, and biomedical sciences. Behav Res Methods. 2007;39(2):175–91. 10.3758/BF03193146. 17695343

[20] Harris R, Ayers S. What makes labour and birth traumatic? A survey of intrapartum ‘hotspots.’ Psychol Health. 2012;27(10):1166–77. 10.1080/08870446.2011.649755. 22292475

[21] Hughes NJ, Namagembe I, Nakimuli A, Sekikubo M, Moffett A, Patient CJ, et al. Decision-to-delivery interval of emergency cesarean section in Uganda: a retrospective cohort study. BMC Pregnancy Childbirth. 2020;20(1):324. 10.1186/s12884-020-03010-x. PMC7251662 32460720

[22] Keten-Edis E, Bal S, Koç E, Başgöl Ş. Determination of Traumatic Birth Perception and Breastfeeding Self-Efficacy Levels of Women Who Had Emergency and Planned Cesarean. Manisa Celal Bayar University Journal of Institute of Health Science. 2023; 10(4), 372–380. 10.34087/cbusbed.1355297

[23] Khsim IEF, Rodríguez MM, Riquelme Gallego B, Caparros-Gonzalez RA, Amezcua-Prieto C. Risk factors for post-traumatic stress disorder after childbirth: a systematic review. Diagnostics (Basel). 2022;12(11):2598. 10.3390/diagnostics12112598. PMC9689389 36359442

[24] Lukasse M, Schei B, Ryding EL. Prevalence and associated factors of fear of childbirth in six European countries. Sex Reprod Healthc. 2014;5(3):99–106. 10.1016/j.srhc.2014.06.007. 25200969

[25] Olsen B, Forgaard A, Nordsletta AS, Sommerseth E, Røseth I. I shut it out”: expectant mothers’ fear of childbirth after a traumatic birth—a phenomenological study. Int J Qual Stud Health Well-being. 2022;17(1):2101209. 10.1080/17482631.2022.2101209. PMC9302015 35852421

[26] Orovou E, Antoniou E, Zervas I. Prevalence and correlates of postpartum PTSD following emergency cesarean sections: implications for perinatal mental health care: a systematic review and meta-analysis. BMC Psychol. 2025;13:26 (**[diğer yazarlar]**). 10.1186/s40359-025-02344-5 PMC11720582 39789649

[27] Ponti L, Smorti M, Ghinassi S, Mannella P, Simoncini T. Can a traumatic childbirth experience affect maternal psychopathology and postnatal attachment bond? Curr Psychol. 2020. 10.1007/s12144-020-00650-2.

[28] Pop-Jordanova N. Childbirth-related psychological trauma. Prilozi (Makedonian Academy of Sciences and Arts, Section of Medical Sciences). 2022;43(1):17–27. 10.2478/prilozi-2022-0002. 35451296

[29] Rashid N, Nalliah S. Understanding the decision-delivery interval in cesarean births. IeJSME. 2007; 1(2): 61–68. 10.56026/imu.1.2.61

[30] Republic of Türkiye Ministry of Health, General Directorate of Health Information Systems. (2025, January 31). Health Statistics Yearbook 2023. https://www.saglik.gov.tr/TR-107086/saglik-istatistikleri-yilligi-2023-yayinlanmistir.html

[31] Rodríguez-Almagro J, Hernández-Martínez A, Rodríguez-Almagro D, Quirós-García JM, Martínez-Galiano JM, Gómez-Salgado J. Women’s perceptions of living a traumatic childbirth experience and factors related to a birth experience. Int J Environ Res Public Health. 2019;16(9):1654. 10.3390/ijerph16091654. PMC6539242 31085980

[32] Royal College of Obstetricians and Gynecologists. Birth after Previous Caesarean Birth (Green-top Guideline No. 45). 2015. https://www.rcog.org.uk/guidance/browse-all-guidance/green-top-guidelines/birth-after-previous-caesarean-birth-green-top-guideline-no-45/

[33] Sigurðardóttir VL, Hákonardóttir GA, Arnardóttir SB, Lýðsdóttir LB, Swift EM. Postpartum post-traumatic stress symptoms and mother–infant bonding: a population-based cross-sectional study. Sex Reprod Healthc. 2025;45:101140. 10.1016/j.srhc.2025.101140. 40845644

[34] Słabuszewska-Jóźwiak A, Szymański JK, Ciebiera M, Sarecka-Hujar B, Jakiel G. Pediatrics consequences of caesarean section-a systematic review and meta-analysis. Int J Environ Res Public Health. 2020;17(21):8031. 10.3390/ijerph17218031. PMC7662709 33142727

[35] Størksen HT, Garthus-Niegel S, Vangen S, Eberhard-Gran M. The impact of previous birth experiences on maternal fear of childbirth. Acta Obstet Gynecol Scand. 2013;92(3):318–24. 10.1111/aogs.12072. 23278249

[36] Şolt-Kırca A, Savaşer S. The effect of birth number on mother-baby attachment. J Health Sci Prof. 2017;4:236–43.

[37] Taheri M, Takian A, Taghizadeh Z, Jafari N, Sarafraz N. Creating a positive perception of childbirth experience: systematic review and meta-analysis of prenatal and intrapartum interventions. Reprod Health. 2018;15:73. 10.1186/s12978-018-0511-x. PMC5932889 29720201

[38] Taylor A, Atkins R, Kumar R, Adams D, Glover V. A new mother-to-infant bonding scale: links with early maternal mood. Arch Womens Ment Health. 2005;8:45–51. 10.1007/s00737-005-0074-z 15868385

[39] Toohill J, Fenwick J, Gamble J, Creedy DK. Prevalence of childbirth fear in an Australian sample of pregnant women. BMC Pregnancy Childbirth. 2014;14:275. 10.1186/1471-2393-14-275. PMC4138382 25123448

[40] Tolja R, Nakić Radoš S, Anđelinović M. The role of maternal mental health, infant temperament, and couple’s relationship quality for mother–infant bonding. J Reprod Infant Psychol. 2020;38(4):395–407. 10.1080/02646838.2020.1733503. 32118491

[41] World Health Organization (WHO). WHO statement on caesarean section rates. 2015. https://www.who.int/news/item/09-04-2015-caesarean-sections-should-only-be-performed-when-medically-necessary

[42] World Health Organization (WHO). WHO recommendations: intrapartum care for a positive childbirth experience. Geneva, Switzerland: World Health Organization; 2018. 30070803

[43] World Health Organization (WHO). Caesarean section rates continue to rise amid growing inequalities in access. 2021. https://www.who.int/news/item/16-06-2021-caesarean-section-rates-continue-to-rise-amid-growing-inequalities-in-acces

[44] Yalnız H, Canan F, Genç RE, Kuloğlu MM, Geçici Ö. Development of a scale of traumatic childbirth perception. Turk Med J. 2016;8(3):81–8.

