# Supplementary material for: The relationship between traumatic birth perception and mother-infant bonding of women who had emergency and planned caesarean sections
Source: BMC Pregnancy Childbirth. 2026 Sep 15;26:1200. doi: 10.1186/s12884-026-09958-6 (PMC13685339; doi:10.1186/s12884-026-09958-6)
Supplement: Supplementary file 1 [file 12884_2026_9958_MOESM1_ESM.pdf]

A. Emergency C/S

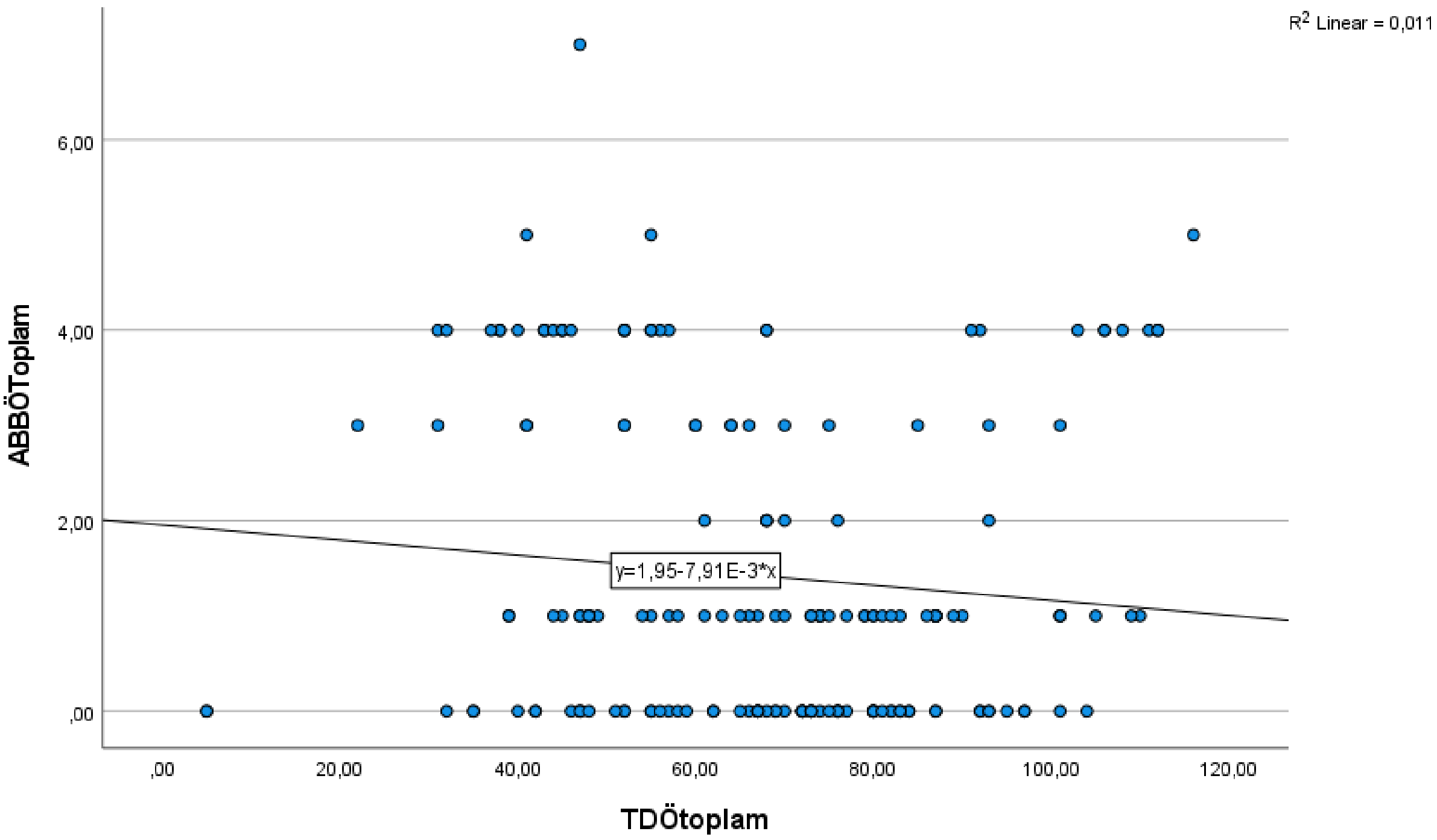

B. Planned C/S

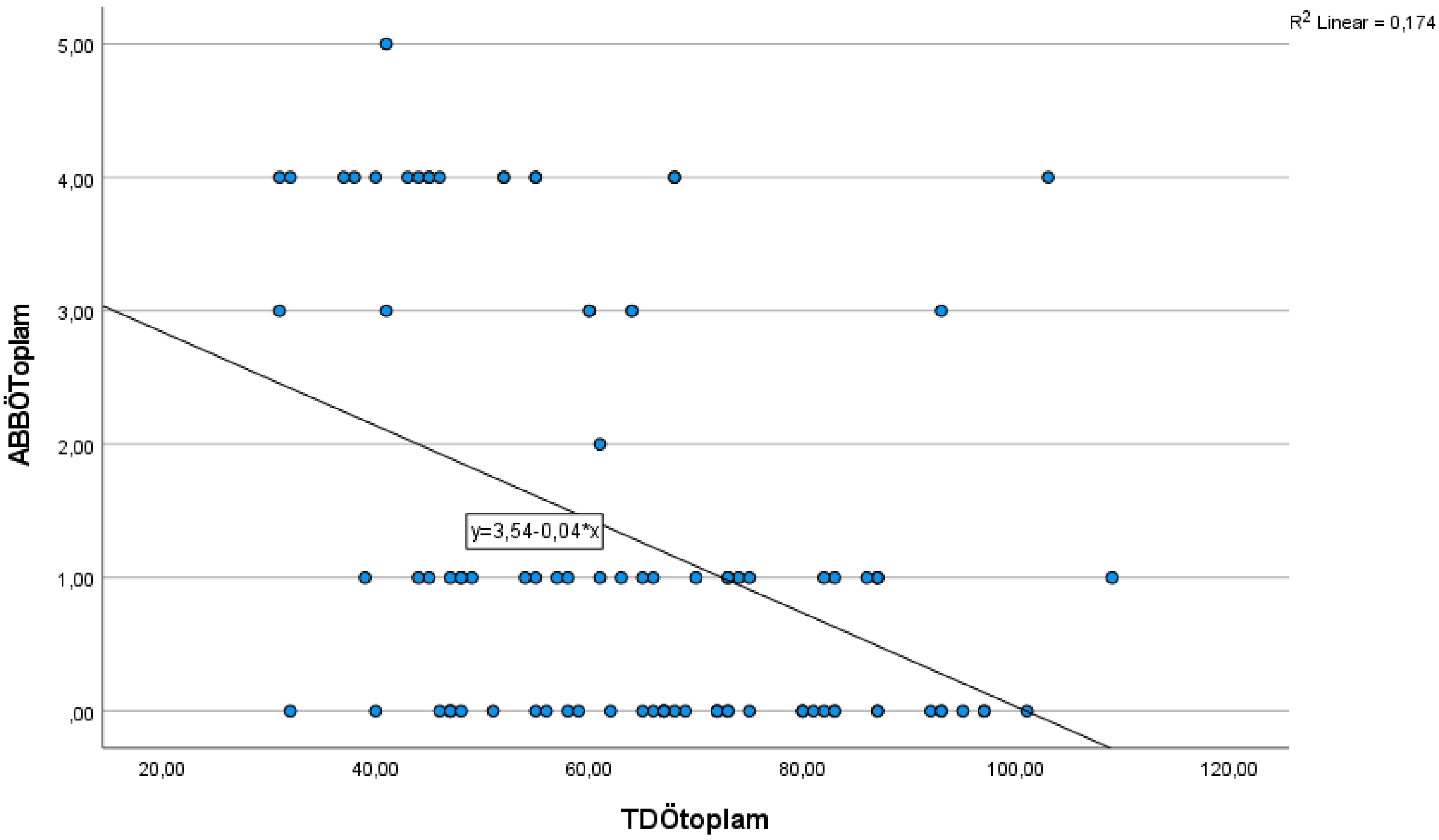

Reviewer-3 comment:

The abstract states that bonding problems decrease as traumatic birth perception increases. However, the Discussion states that attachment weakens as traumatic birth perception increases. These interpretations directly contradict each other. Correct the abstract, Results, Discussion, and Conclusion based on the verified direction. Include a scatterplot of TCPS and MIBS scores for each caesarean group, with an appropriate fitted trend and confidence interval. Test a TCPS-by-caesarean-group interaction in a regression model, or formally compare the two correlation coefficients.

Response:

We thank the reviewer for identifying this important issue. We rechecked the scoring procedure of the Mother to Infant Bonding Scale (MIBS). As described in the Methods section, higher MIBS scores indicate greater mother–infant attachment difficulties. Therefore, the negative correlation observed between traumatic childbirth perception and MIBS scores in the planned cesarean group indicates that higher traumatic childbirth perception scores were associated with lower reported attachment difficulty scores. The interpretation of this finding has been revised throughout the Abstract, Results, Discussion, and Conclusion sections to avoid an incorrect directional interpretation and causal inference.

Additionally, scatterplots with fitted linear trend lines were generated separately for emergency and planned cesarean groups to visually examine the association between traumatic childbirth perception and mother–infant attachment scores (Supplementary Figure 1). The scatterplots demonstrate the absence of a statistically significant linear association in the emergency cesarean group and a negative association pattern in the planned cesarean group, consistent with the correlation analyses.

Supplementary Figure 1. Scatterplots showing the relationship between traumatic childbirth perception and mother–infant attachment problem scores according to cesarean section type.

A: Emergency cesarean section group.

B: Planned cesarean section group.

Higher MIBS scores indicate greater mother–infant attachment difficulties. Linear fitted lines are presented to demonstrate the direction of the observed associations.
